# Nucleotide-dependent DNA gripping and an end-clamp mechanism regulate the bacteriophage T4 viral packaging motor

**DOI:** 10.1038/s41467-018-07834-2

**Published:** 2018-12-21

**Authors:** Mariam Ordyan, Istiaq Alam, Marthandan Mahalingam, Venigalla B. Rao, Douglas E. Smith

**Affiliations:** 1 Department of Physics, University of California, San Diego, 9500 Gilman Drive, Mail Code 0379, La Jolla, CA 92093-0379 USA; 2 Department of Biology, The Catholic University of America, 620 Michigan Ave. NE, Washington, DC 20064 USA

## Abstract

ATP-powered viral packaging motors are among the most powerful biomotors known. Motor subunits arranged in a ring repeatedly grip and translocate the DNA to package viral genomes into capsids. Here, we use single DNA manipulation and rapid solution exchange to quantify how nucleotide binding regulates interactions between the bacteriophage T4 motor and DNA substrate. With no nucleotides, there is virtually no gripping and rapid slipping occurs with only minimal friction resisting. In contrast, binding of an ATP analog engages nearly continuous gripping. Occasional slips occur due to dissociation of the analog from a gripping motor subunit, or force-induced rupture of grip, but multiple other analog-bound subunits exert high friction that limits slipping. ADP induces comparably infrequent gripping and variable friction. Independent of nucleotides, slipping arrests when the end of the DNA is about to exit the capsid. This end-clamp mechanism increases the efficiency of packaging by making it essentially irreversible.

## Abstract (web summary)

Packaging of viral DNA depends on strong molecular motors that are powered by ATP hydrolysis. Here, the authors develop a single-molecule assay to monitor how nucleotide binding regulates motor-DNA interactions and reveal a generic mechanism that prevents exit of the whole DNA from the viral capsid during packaging.

## Introduction

Many double-stranded DNA viruses follow a remarkable assembly pathway in which empty viral capsid shells assemble first and the viral genome is then packaged into them by a molecular motor via a portal nanochannel having a diameter of ~3.5–4 Å^1–6^. The process of confining the DNA to a high density, approaching that of a crystalline solid, is energetically unfavorable due to electrostatic self-repulsion of negatively charged DNA segments, DNA bending rigidity, and entropy loss^7–11^. A strong molecular motor, powered by ATP hydrolysis, is needed to accomplish this process. Viruses that employ this assembly mechanism include bacteriophages, as well as many viruses that infect humans, including herpesviruses, adenoviruses, and poxviruses. Viral packaging motors are also related to many other cellular molecular motors, including helicases, chromosome transporters, and protein translocases^12,13^.

In prior work, we developed techniques using optical tweezers to measure the packaging of single DNA molecules and applied them to study phages phi29, T4, and lambda. We found that the motors generate high forces (>60 pN) and translocate DNA at speeds ranging from ~100 bp s^−1^ to ~2000 bp s^−1^, depending on ATP concentration, ionic conditions, and temperature^14–19^. The motors are ring-shaped, having multiple subunits each with the capacity to hydrolyze ATP, and the DNA is constrained to pass through the center channel of the ring. Structural data suggests there are five subunits^20–22^. Current models propose that motor subunits grip DNA one at a time and that the interactions of other subunits with the DNA exert no influence on the motion^20,21,23,24^, but the latter hypothesis has not been tested.

Gripping of the DNA by one subunit at a time has been proposed as the simplest mechanism because it avoids the problem that a given subunit could have trouble translocating the DNA if other subunits were simultaneously gripping^25^. It has also been pointed out that because the pitch of the DNA double helix is ~10.5 bp per turn each subunit of a pentameric motor ring would have a unique alignment with the threaded DNA^20^. A model based on structural data for T4 proposes that positive charged residues in the aligned subunit grip the negatively charged DNA phosphates via electrostatic interactions^20^. After ATP hydrolysis the DNA is suggested to be translocated by 2 bp, such that the next subunit in the ring would approximately come into alignment with the phosphate backbone.

Several studies have provided evidence suggesting that ATP binding causes the motor protein to undergo a conformational change that enables it to grip DNA tightly. One piece of evidence is that when the [ATP] is lowered, slipping occurs more frequently^24–26^. Another piece of evidence is that pauses in translocation are induced when a small amount of a non- (or slowly) hydrolyzable ATP analog is mixed with ATP. This has been interpreted as gripping of the DNA by an analog-bound subunit^24,25^.

The phi29 motor has been extensively studied^14,17,23,27,28^ and measurements suggest that one special subunit aligns with the phosphate backbone and grips it tightly. After all five subunits bind ATP, the special subunit hydrolyzes ATP and releases its grip. Then, in a highly coordinated fashion, the other subunits sequentially each hydrolyze ATP, release P_i_, translocate DNA by 2.5 bp, and release ADP. However, it is not clear how DNA gripping interactions are regulated when multiple subunits have ATP bound. It is also not clear whether the model described for phi29 applies universally to other viral motors. Recent studies found evidence that the T4 motor subunits are not strictly coordinated^29^. Phylogenetic analyses also suggest that viral motors classified in the terminase superfamily of ASCE ATPases, such as the T4 motor, evolved independently from the phi29 motor, which is classified in the HerA/FtsK superfamily^30^.

We found that the T4 and lambda terminase motors translocate much faster than the phi29 motor and exhibit differences in pausing and slipping^16^. Also, when slips occur during T4 packaging the DNA moves much more slowly than when slips occur during phi29 packaging^16,24^. Studies with varying [ATP] and mixtures of ATP and non- (or slowly) hydrolyzable analogs led to a model which proposed that the T4 motor pauses when a subunit in the apo state (with no nucleotide) grips the DNA weakly and enters an off pathway “unpackaging” state^24^. Pausing was proposed to allow ATP binding and unpackaging was proposed to allow the motor to re-establish correct alignment with the DNA.

Here, we introduce an assay that provides insights into the regulation of the phage T4 motor’s grip and kinetic friction between motor subunits and the DNA. Our measurements directly and unambiguously show that the T4 motor has strongly persistent grip on DNA in the ATP-bound state, intermittent grip in the ADP-bound state, and virtually no grip in the apo state. The apo state measurements quantify a minimum friction level that limits the rate of DNA movement out of the viral capsid. Our measurements also show that multiple nucleotide-bound subunits interact simultaneously with the DNA and exert friction that limits slipping. Finally, we reveal a unique “end clamp” mechanism that prevents the whole DNA from exiting the motor channel even if the motor slips.

## Results

### Motor grip assay

To investigate how the motor’s grip on DNA is regulated, we combined single DNA molecule manipulation with optical tweezers^31,32^ and rapid microfluidic solution exchange. These techniques have been combined previously in, for example, studies of RecBCD helicase/nuclease^33^. Here, we introduce an assay in which DNA packaging is first initiated with ATP and, after proceeding for a defined time, the actively packaging complex is then suddenly moved out of ATP and into solutions containing either no nucleotides, high-concentrations of a non- (or slowly) hydrolyzable ATP analog (γ-S-ATP), or the hydrolysis product, ADP. This allows us to characterize the nature of the motor-DNA interactions in defined conditions where motor subunits are switched to a single state: apo, ATP analog-bound, or ADP-bound, respectively. Packaging stops and the persistence of motor’s grip in each state is quantified.

**Fig. 1:**
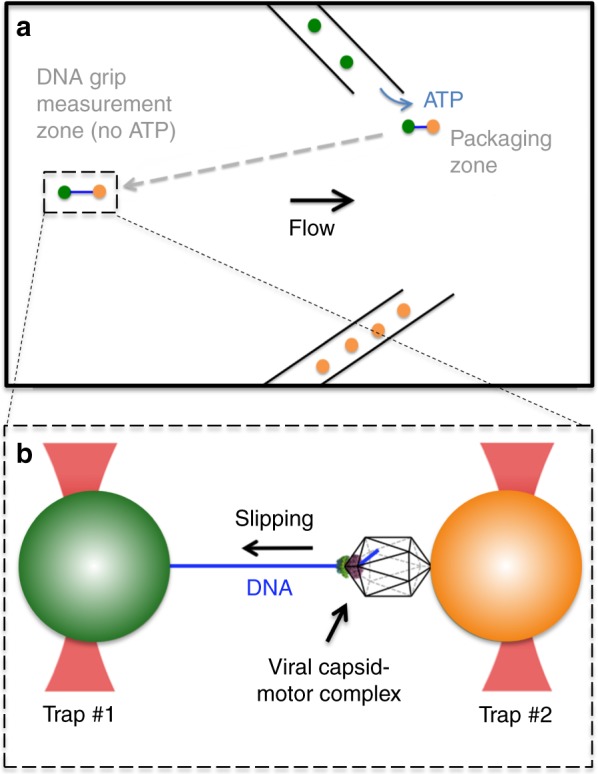
Schematic illustration of the experiment. **a** Microspheres coated with capsid-motor complexes (orange) are dispensed by a micro-capillary tube into a fluid chamber. One of these microspheres is trapped by an optical tweezer beam and is brought near a second micro-capillary tube that is dispensing ATP and microspheres coated with DNA (green). **b** One of these DNA microspheres is trapped with a second tweezer beam and brought near the capsid-motor complexes to initiate packaging. After ~4–7 kbp of DNA (blue) is packaged the complex is rapidly moved out of ATP (away from the micro-capillary tube, as shown by the dashed arrow) and into a region of the chamber (the “grip measurement zone”) containing either no nucleotides, γ-S-ATP, or ADP. A small stretching force (3–5 pN) is applied to the DNA while the motor’s grip is monitored

Measurements are conducted as shown in Fig. 1. Microspheres coated with capsid-motor complexes are dispensed by a micro-capillary tube into a microfluidic chamber. One microsphere is trapped by a focused laser beam and brought near a second micro-capillary dispensing DNA-coated microspheres and ATP. One of the DNA microspheres is trapped with a second focused laser beam and brought near the capsid-motor complexes to initiate packaging. A small tension of ~3–5 pN is applied to stretch the unpackaged section of DNA so that its length can be accurately measured^14,16,34,35^.

If translocation is detected, it is allowed to continue until ~4–7 kbp of DNA is packaged. This is a small fraction of the genome length (~171 kbp), so internal forces resisting packaging are expected to be negligible^28,36,37^. The complex is then quickly moved (within ~1 s) into a region of the chamber containing either no nucleotides, γ-S-ATP, or ADP. A gentle flow ensures that ATP does not enter this region. High concentrations (0.5 mM) of γ-S-ATP or ADP are used such that these nucleotides are expected to displace all the ATP bound to the motor subunits. The motor translocates DNA at ~600 bp s^−1^ with 0.5 mM ATP^16,38^. Since structural data suggests ~2 bp are translocated per ATP hydrolyzed^20^, each iteration of the hydrolysis-translocation cycle must take only ~2/600 = 1/300th of a second. Therefore, hydrolysis of residual ATP and binding of added nucleotides is expected to occur very rapidly before the measurement starts.

### The nucleotide state of motor subunits controls DNA gripping

**Fig. 2:**
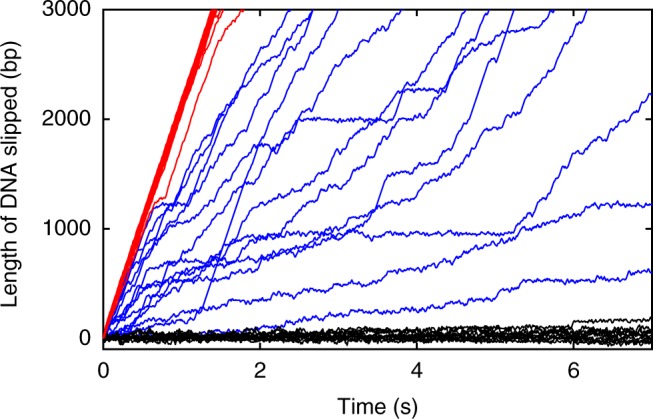
Measurements of DNA gripping by the motor and slipping. Representative measurements of length of DNA that slips out of the capsid vs. time with no nucleotides (red lines, average force = 3 pN), 0.5 mM γ-S-ATP (black lines, average force = 5 pN), or 0.5 mM ADP (blue lines, average force = 5 pN). Each line is a separate slipping event. Note that many red lines lie nearly on top of each other, and similarly for the black lines. The total numbers of solution exchange events and complexes measured for each condition, including ones not plotted here, are given in the results section of the text

The measurements show that the T4 motor spends drastically different fractions of time gripping the DNA depending on the nucleotide state. Throughout the paper we use the term “gripping” to mean periods of time when there is no detected slipping (i.e., movement of the DNA out of the capsid). With no nucleotides there is almost no gripping and the DNA slips very fast (usually ~2000 bp s^−1^, *N*_m_ = 25 solution exchange measurements on *N*_c_ = 15 complexes) (Fig. 2). In contrast, with 0.5 mM γ-S-ATP the DNA slips only a small fraction of the time and the motor grips most of the time. The net rate of DNA movement, averaged over all time (during both gripping and slipping), is $\bar v = 2.3\,{\mathrm{bp}}\,{\mathrm{s}}^{ - 1}$ (standard error in the mean (SEM) = 0.54, *N*_m_ = 9, *N*_c_ = 9), which is ~1000× lower than with no nucleotide. These findings directly and unambiguously show that motor subunits almost never grip when the ATP binding pocket is empty and can grip persistently when the ATP analog is bound. Interestingly, when ADP is used highly intermittent gripping is observed. Slipping is much more frequent than with γ-S-ATP but less frequent than with no nucleotide. The net rate of DNA movement is $\bar v = 844\,{\mathrm{bp}}\,{\mathrm{s}}^{ - 1}$ (SEM = 93; *N*_m_ = 35, *N*_c_ = 16), which falls between that measured with no nucleotide and with γ-S-ATP.

**Fig. 3:**
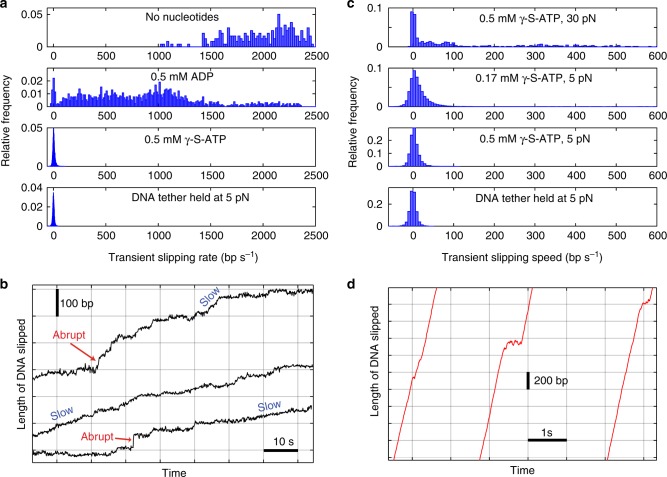
DNA slipping velocities and transient slipping events. **a** Distributions of slipping velocities measured in 1 s time intervals in the four different conditions indicated in the plot legends. “DNA tether held at 5 pN” indicates results from control experiments with fixed-length tethered DNA molecules (with no capsid motor complexes) to characterize transient non-zero velocities measured due to Brownian fluctuations and instrument noise. **b** Examples of different velocities of slipping (rare “abrupt” slips and more common “slow” slips) measured with 0.5 mM γ-S-ATP. Each plotted line is a single event, arbitrarily positioned on the graph, with length and time scales indicated by the scale bars. **c** Distributions of slipping velocities measured in 1 s time intervals for the four different conditions indicated in the plot legends. **d** Examples of brief decelerations during slipping which are observed infrequently in absence of nucleotides. Each plotted line is a single event, arbitrarily positioned on the graph, with length and time scales indicated by the scale bars

To further quantify the gripping/slipping dynamics we calculated the distribution of transient velocities recorded in each condition (Fig. 3a). With no nucleotide, the velocity is almost always >1500 bp s^−1^ and ranges as high as ~2500 bp s^−1^. With 0.5 mM ADP, it ranges from 0 to ~2500 bp s^−1^ because there is a mixture of gripping and variable-speed slipping. With 0.5 mM γ-S-ATP, it is usually near zero, indicating that the motor is usually gripping the DNA. Below we discuss each of these conditions in more detail.

### Motor grip with γ-S-ATP bound

When 0.5 mM γ-S-ATP is added, the motor grips most of the time. A small amount of slipping occurs, as shown in Fig. 3b, but the slips result in only a small amount of net DNA movement over time $\left( {\bar v = 2.3\,{\mathrm{bp}}\,{\mathrm{s}}^{ - 1}} \right)$, which is negligible compared with the ~600 bp s^−1^ packaging rate with 0.5 mM ATP. Short, abrupt slips (~20–50 bp in <1 s) are occasionally observed (Fig. 3b), but only 13 were detected during a total measurement time of 855 s and they added up to only 6.6 s (~0.8% of the total time). Longer, slower slips are more frequent and are characterized by the transient velocity distribution (Fig. 3c).

Because Brownian fluctuations and instrument noise influence the measurements, we first conducted control experiments with a DNA molecule alone (with no motor complex) tethered between two microspheres^39^ to quantify the minimum detectable slipping velocity (bottom panel, Fig. 3a,c). As expected, the average velocity in 1 s intervals is close to zero (0.002 bp s^−1^), but a standard deviation (SD) *σ*_control_ = 8.3 bp s^−1^ characterizes the effects of Brownian fluctuations and instrument noise.

For the motor complexes with γ-S-ATP, 16% of 1 s intervals exhibit velocities ≥2*σ*_control_, which indicates that slipping occurs ~16% of the time. The slipping velocity (DNA movement rate during slipping) averages $\bar v_{\mathrm{s}} = 33.8\,{\mathrm{bp}}\,{\mathrm{s}}^{ - 1}$ (SD = 18.9). There is no systematic increase in the velocity during the slips, which implies that a 5 pN kinetic friction force, on average, is exerted by the motor on the DNA and opposes the 5 pN applied force. Strikingly, the average slipping velocity is ~60× lower than that measured with no nucleotides. This shows that during these slips γ-S-ATP remains bound to some subunits, because dissociation from all subunits would result in slipping at ~2000 bp s^−1^. That the average slipping velocity is ~60× lower than that measured without nucleotides, despite similar applied force indicates that the friction is velocity dependent and that motor subunits with γ-S-ATP-bound exert much stronger friction on the DNA than apo subunits.

To investigate what causes loss of motor grip we varied γ-S-ATP concentration and applied force (Fig. 3c). We first kept the force at 5 pN and modestly lowered γ-S-ATP from 0.5 to 0.17 mM. During packaging with hydrolyzable ATP, reducing [ATP] from 0.5 to 0.17 mM reduces the motor velocity only slightly since these concentrations are in the near-saturating range^24^. For either concentration, since the estimated time it takes γ-S-ATP to dissociate (~1 s)^24^ is much longer than the time it takes to bind (<1/300 s), all five subunits would usually have γ-S-ATP bound. However, we found that decreasing γ-S-ATP from 0.5 to 0.17 mM increases $\bar v$ from 2.3 bp s^−1^ to 14.5 bp s^−1^ (SD = 10.5 bp s^−1^; *N*_m_ = 17, *N*_c_ = 15) and increases the fraction of 1 s intervals that exhibit slipping from 16% to 31.6%. This suggests that with 0.17 mM γ-S-ATP many of the slips are attributable to transient dissociation of γ-S-ATP.

**Fig. 4:**
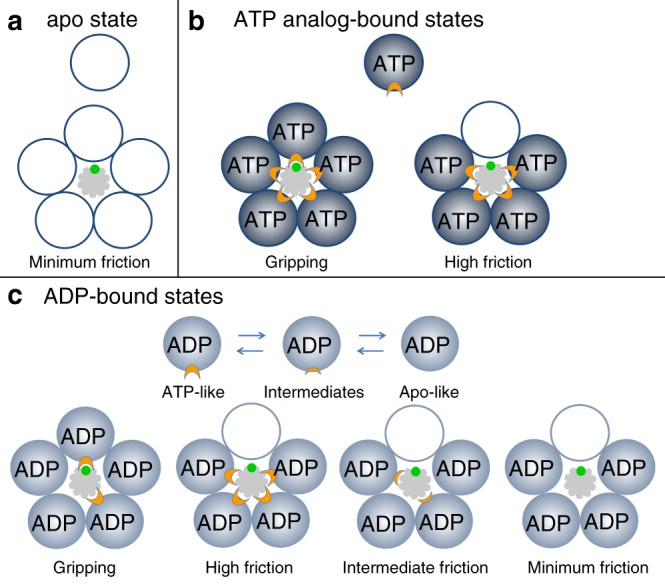
Model for motor-DNA interactions. **a** In the apo state motor subunits (depicted as five empty circles) do not grip the DNA (gray). They exert only minimal friction, and the DNA slips rapidly. **b** With the ATP analog bound the subunits (blue circles; the label “ATP” refers to the analog γ-S-ATP) are in a gripping-capable conformation, but only one subunit is optimally aligned to form a grip, consistent with structural data^20^. If the γ-S-ATP dissociates the grip is released, but the other subunits still have γ-S-ATP bound and are in strong frictional contact, resulting in very slow slipping. The region of the motor protein that grips the DNA is schematically indicated by the orange “gripper” and the region of the DNA structure that is gripped is schematically indicated as a green “patch”. Specific regions involved have been proposed based on structural studies^20, 21, 51^. **c** With ADP bound the motor subunits (labeled circles) fluctuate between a gripping/high friction conformation similar to the ATP-bound state, intermediate-friction conformations (schematically indicated by smaller orange “grippers”), and a minimum friction state similar to the apo state. If ADP dissociates from a gripping subunit the grip is released, but the other subunits exert friction on the DNA. The amount of friction can vary widely depending on how many subunits are in ATP-like, intermediate, or Apo-like conformations

The average slipping velocity ($\bar v_{\mathrm{s}} = 39.2\,{\mathrm{bp}}\,{\mathrm{s}}^{ - 1}$; SD = 35.6 bp s^−1^) measured with 0.17 mM γ-S-ATP is similar to that measured with 0.5 mM γ-S-ATP ($\bar v_{\mathrm{s}} = 33.8\,{\mathrm{bp}}\,{\mathrm{s}}^{ - 1}$; SD = 18.9), which suggests that roughly the same number of subunits, on average, exert friction on the DNA in either condition. Our interpretation is that one subunit grips the DNA and the other four subunits are in frictional contact. Slips occur when γ-S-ATP transiently dissociates from the gripping subunit (see model, Fig. 4) and more slipping occurs with the lower 0.17 mM concentration because the γ-S-ATP takes longer to rebind after dissociating (Fig. 4b). This would occur whether γ-S-ATP dissociates or whether it is first hydrolyzed and then the products dissociate. This conclusion is consistent with structural studies of the T4 motor which suggest that only one subunit at a time is properly aligned to grip the DNA^20^.

We next kept γ-S-ATP at 0.5 mM and increased the force to 30 pN, the estimated maximum resisting packaging^8,28,37^. This causes a large increase in $\bar v$ from 2.3 bp s^−1^ to 166 bp s^−1^ (SD = 252 bp s^−1^, *N*_m_ = 3, *N*_c_ = 3) and increases the fraction of intervals that exhibit slipping from 16% to 60.1%. Thus, with the 30 pN force, most of the slipping is attributable to force-induced rupture of the motor’s grip. The slipping velocity averaged $\bar v_{\mathrm{s}} = 275\,{\mathrm{bp}}\,{\mathrm{s}}^{ - 1}$ (SD = 275 bp s^–1^), which is ~10-fold higher than with the 5 pN force. There is no systematic increase in the velocity during the slips, which indicates that a 30 pN kinetic friction force, on average, opposes the 30 pN applied force. That higher force causes faster slipping further confirms that the friction force is velocity dependent. We note that although the 30 pN force causes significant slipping it does not prevent the motor from functioning. With saturating [ATP] the DNA is still packaged at a net rate of ~200 bp s^−1^^38^. The average motor velocity (rate of packaging not including slips) is ~350 bp s^−1^, which is higher than the backwards rate of DNA movement due to slipping measured here with γ-S-ATP $\left( {\bar v = 166\,{\mathrm{bp}}\,{\mathrm{s}}^{ - 1}} \right)$.

It is possible that γ-S-ATP preparations could contain stray ADP. Since we observe frequent slipping with ADP, it is possible that some of the slips observed with γ-S-ATP could be due to ADP binding. However, the increase in slipping upon dilution of γ-S-ATP implies the increased slipping with 0.17 mM γ-S-ATP is due to dissociation of γ-S-ATP, not increased ADP binding, because any ADP present in the γ-S-ATP preparation would be diluted along with the γ-S-ATP. The increase in slipping with increasing applied force is also not attributable to ADP because the concentration of γ-S-ATP, and any stray ADP, was kept constant.

### Motor grip with no nucleotides

In the absence of nucleotides, the DNA slips very rapidly (Figs. 2 and 3a) and to track it the optical trap had to be moved at the maximum speed our instrument allows (~730 nm s^−1^). The average slipping velocity is $\bar v_{\mathrm{s}} = 2000\,{\mathrm{bp}}\,{\mathrm{s}}^{ - 1}$ (SD = 350 bp s^−1^) and the applied force averages 3 pN (SD = 1.6). There is no systematic increase in the velocity during slips, which implies that a kinetic friction force of 3 pN, on average, opposes the 3 pN applied force. Since the fastest slipping occurs when all motor subunits are in the apo state, this measurement quantifies a “speed limit” for how fast the DNA can move out of the capsid and through the portal and motor channels, when driven by a small force of 3 pN. In fact, the slipping velocity in different time intervals varies from ~1000–2500 bp s^−1^, suggesting that the level of friction fluctuates. In any case, the influence of friction exerted by subunits in the apo state (Fig. 4a) is minimal compared with that exerted by subunits in the γ-S-ATP-bound state. In general, sources of friction would include interactions between the DNA and the motor ring, the portal channel, and the capsid walls, as well as hydrodynamic drag, and self-friction between DNA segments inside the capsid.

The velocity distribution does not exhibit any 1 s intervals having near-zero velocities consistent with gripping events, but brief decelerations were occasionally resolved on shorter time scales. Examples of such events are shown in Fig. 3d. These were detected only ~2% of the time and their average duration is 0.08 s (SD = 0.07; *n* = 40 events). These events may indicate that motor subunits occasionally fluctuate into a state where they briefly grip or exert friction on the DNA despite not having ATP bound. However, another possibility is that they occur due to transient jamming of tangled or knotted sections of the DNA inside the capsid^40–43^. In any case, these events are so infrequent that they have a negligible effect on the average slipping velocity.

### Motor grip with ADP bound

During normal motor operation each subunit cycles through a third state where, after binding ATP and hydrolyzing it, the binding pocket is occupied (transiently) by an ADP molecule^23^. To investigate the nature of motor-DNA interactions with ADP bound we conducted measurements with high ADP (0.5 mM). Gripping is observed (Fig. 2), but in only 4.6% of time intervals, which is ~18-fold fewer than with 0.5 mM γ-S-ATP. The slipping velocity is lower than measured with no nucleotides, averaging $\bar v_{\mathrm{s}} = 885\,{\mathrm{bp}}\,{\mathrm{s}}^{ - 1}$ (SD = 532 bp s^−1^), which implies that some motor subunits are in a conformation where they exert friction on the DNA. However, the slipping velocity is ~26-fold higher, on average, than measured with 0.5 mM γ-S-ATP, which implies that the friction is much smaller.

Modest dilution of ADP from 0.5 to 0.17 mM decreases the fraction of time the DNA is gripped from 4.6% to 2.3% (*N*_m_ = 30, *N*_c_ = 21). Thus, with 0.17 mM ADP, much of the slipping is attributable to transient dissociation of ADP. However, the average slipping velocity ($\bar v_{\mathrm{s}} = 874\,{\mathrm{bp}}\,{\mathrm{s}}^{ - 1}$; SD = 598 bp s^−1^) with 0.17 mM ADP is consistent with that observed with 0.5 mM ADP. This implies that roughly the same number of subunits, on average, exert friction in either condition. Similar to the case with γ-S-ATP, this suggests that in either condition all five subunits normally have ADP bound, one subunit at a time grips the DNA, and slipping occurs when ADP dissociates from that subunit (Fig. 4c). The other four subunits would usually have ADP bound and exert friction on the DNA. The gripping conformation with ADP bound could be similar to that with ATP bound, and for simplicity is depicted that way in Fig. 4, but need not be exactly the same. With the lower 0.17 mM concentration ADP rebinding following dissociation would be slower, resulting in a lower % time gripping. The much higher % time gripping measured with γ-S-ATP is attributable to much slower dissociation of γ-S-ATP than ADP and a more stable gripping conformation. However, a distinct difference in the behavior with ADP is that the slipping velocity is highly variable, ranging from as low as that observed with γ-S-ATP $\left( {\bar v_{\mathrm{s}} = 34\,{\mathrm{bp}}\,{\mathrm{s}}^{ - 1}} \right)$ to as high as that observed with no nucleotide $\left( {\bar v_{\mathrm{s}} = 2000\,{\mathrm{bp}}\,{\mathrm{s}}^{ - 1}} \right)$. Our interpretation of this finding is that ADP bound subunits can fluctuate into multiple different conformations which either grip DNA or exert variable levels of friction (Fig. 4c). Such conformations may be intermediates between an ATP-bound-like (high friction) conformation and apo-like conformation (minimum friction).

Further analyses of the 0.5 mM ADP data are presented in Supplementary Note 1 and stochastic simulations of the model (Fig. 4c) are presented in Supplementary Note 2. We find that the distributions of durations of the slipping and gripping events (Supplementary Figs. 1 and 2) are consistent with the model and determine rate constants for transitions between the gripping and slipping states. The simulations show that the predictions of the model are consistent with the experimental findings (Supplementary Fig. 3).

The observation that gripping events can last >1 s implies that, under the conditions of the assay, ADP can take >1 s to dissociate and when a subunit grips DNA the gripping conformation is stabilized. During normal motor operation, when bound ADP is generated as the hydrolysis product of ATP, the measured maximum translocation velocity implies that ADP is released much faster, within 1/300 s. Our findings therefore suggest that the presence of ATP accelerates the release of ADP. This finding is consistent with a proposed model for the phi29 motor, supported by experiments, in which ATP binding to one subunit induces ADP release from the adjacent subunit^23^.

### An end-clamp mechanism prevents DNA exit

**Fig. 5:**
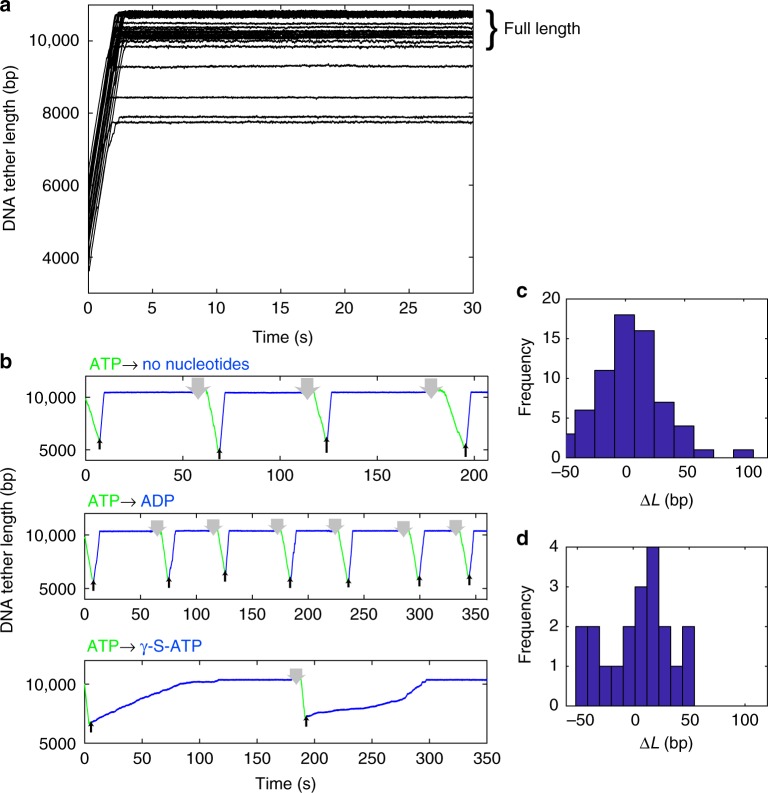
Arrest of slipping at the end of the DNA template. **a** Representative examples of measured DNA tether length vs. time with no nucleotides (28 events). The slipping is initially very fast but it suddenly arrests and the final DNA tether lengths are mostly around the full DNA length (~10 kb). **b** DNA tether length vs. time measurements on single complexes with low (5 pN) applied force where the complex is exposed to ATP, packaging occurs (green lines), and then it is moved out of ATP (blue lines) and into a solution with either no nucleotide, 0.5 mM ADP, or 0.17 mM γ-S-ATP, as indicated in each plot title. When out of ATP rapid slipping occurs, but it arrests at ~10 kbp. After waiting ~60 s to assess the stability of the arrested state the complex is moved back into ATP (green lines) and packaging resumes. The upward arrows indicate times when the complex was moved out of ATP and the downward arrows indicate times when it was moved back into ATP. The process of DNA slipping, arrest, and restarted packaging can be repeated many times. **c** Histogram of measured DNA length differences Δ*L* between arrest lengths (plateaus in **a**) for each complex. **d** Histogram of Δ*L* values measured in control experiments in which arrested complexes were moved in the chamber but did not restart

Although the DNA usually slips rapidly when motor subunits are in the apo or ADP state, we observed a striking exception. After about 4–7 kbp of the DNA slips out, i.e., the length of DNA that was initially packaged in the assay, slipping suddenly arrests and the DNA is prevented from completely exiting the capsid-motor complex. Plots of the DNA tether length (unpackaged length) vs. time (Fig. 5a) show that, regardless of the length of DNA initially packaged in each complex, the ending tether length is around 10 kb, which is the full length of the DNA template. A few complexes stop at ~8–9 kbp, but the majority stop at ~10 kbp (23 out of 27). Thus, a special mechanism must prevent the first-packaged end of the DNA from slipping out of the capsid-motor complex.

Although there is some variation in the final DNA tether length where slipping arrests, the data are consistent with clamping of the DNA at or near its end. The measured variations in final tether length are attributable to variations in the sizes of the microspheres and substrate DNA molecules. The microspheres are nominally 2.2 μm in diameter but vary by ~300 nm. The length of DNA template is nominally 10,051 bp but varies because the molecules were generated by PCR and there was some non-specific priming resulting in amplification of other lengths. To characterize the overall variability, we conducted control experiments in which DNA molecules alone were tethered^39^ and found that the standard deviation in measured lengths is 270 bp. This accounts for most of the variation in final tether lengths in Fig. 5a.

We further found that, after a complex has reached the end-clamped state in the absence of ATP, packaging can be restarted by moving the complex back into the region of the microfluidic chamber containing ATP. In fact, as shown in Fig. 5b, the process of packaging, slipping, end clamping, and re-packaging can be repeated multiple times. Individual runs of slipping/gripping events following solution exchange are stochastic, but no obvious differences in behavior were observed when repeating the measurements on a single complex.

Slipping also arrests in conditions where ADP or γ-S-ATP are present (Fig. 5b). In each case the motor restarts when the complex is moved back into ATP. Thus, the clamp mechanism does not depend on the nucleotide state of the motor proteins. We attempted slipping measurements 204 times and every time the slipping arrested. Thus, the clamped state is very stable. After arrest we waited 60 s and ~95% of the time the DNA did not detach. Control experiments further showed that the occasional loss of the tethered complex is attributable to rupture of the antibody-capsid connection^39^ as opposed to release of the DNA.

Resumption of packaging after end-clamping is efficient. Out of 100 attempts, packaging restarted 78 times. We observed cases where individual complexes could be restarted up to 7 times (Fig. 5b). In these cases, a striking feature is that the DNA repeatedly slips out to almost exactly the same position (Fig. 5c, d). The average difference in repeated clamp positions was only 4 bp, indicating that the DNA is clamped at virtually the exact same position after each slip, presumably at its end. The standard deviation in measured differences of 28 bp is attributable to instrument drifts which occur when the traps are moved into and out of the ATP containing regions. Control experiments with complexes that did not resume packaging, so the tether length stayed constant, yielded a similar standard deviation of 30 bp (Fig. 5d).

## Discussion

Our measurements using the gripping/slipping assay introduced here provide several insights into the regulation of DNA gripping by a viral packaging motor, a basic question that has remained refractory thus far. Our findings lead us to propose a model (Fig. 4) to describe DNA-motor interactions that has implications for the mechanism of DNA packaging.

In the apo state, no motor subunits grip the DNA and rapid slipping occurs with minimum friction (Fig. 4a). Binding of the ATP analog (γ-S-ATP) induces DNA gripping (Fig. 4b). Slipping can occur due to dissociation of the nucleotide or force-induced rupture of the grip. However, ATP-analog bound subunits exert a large amount of friction on the DNA that strongly limits how far the DNA can slip out of the capsid. This is a significant finding since prior models assumed that only one subunit interacts with DNA at a time and the possibility that one or more subunits could exert friction on the DNA had not been considered.

Despite the average pulling force being lower in the no nucleotide measurements than in the γ-S-ATP measurements (3 vs. 5 pN), the slipping velocity is significantly higher. Average slipping velocity with ADP is also higher than with γ-S-ATP despite the pulling force being the same (5 pN). Thus, friction has the strongest influence with γ-S-ATP, weaker influence with ADP, and the weakest with no nucleotides. Our interpretation is that friction force increases with increasing velocity, such as occurs with hydrodynamic drag. We presented evidence for this in the γ-S-ATP measurements in which slipping velocity increased when pulling force was increased from 5 pN to 30 pN. Our interpretation is that when a given force is applied the velocity transiently increases (on a timescale too fast to measure) until the friction force is equal to the applied force and a terminal velocity is reached that depends on the force. In the simplest model Friction Force = (Friction Coefficient) × (Velocity); although in general force could be a nonlinear function of velocity. In this model, our results would imply that the Friction Coefficient is highest with γ-S-ATP, lower with ADP, and lowest with no nucleotides.

Our data suggests that friction exerted by the motor on the DNA that opposes slipping is critical for motor function. With saturating [ATP], it takes ~1/300 s for a subunit to cycle through binding ATP, gripping DNA, hydrolyzing ATP, translocating, releasing ADP, and releasing its grip. With lower [ATP] the cycle time would be longer. If one subunit released its grip and it took 1/300 s for another subunit that is properly aligned with the DNA to cycle into the gripping state, significant slipping could occur. If slipping occurred at the 2000 bp s^−1^ rate characteristic for the apo state, the DNA would slip ~7 bp in 1/300 s, which is several-fold greater than the predicted translocation step size. However, since other ATP-bound subunits exert significant friction then, as we have shown, the slipping velocity is reduced to ~34 bp s^−1^ on average. Thus, the length of a slip would be limited to ~0.1 bp on average in 1/300 s, which is negligible compared to the translocation step size.

Previous studies suggest that T4 motor subunits frequently become misaligned with the DNA and this disrupts the motor’s grip^24^. In addition, it was recently reported that the T4 motor with one or two inactive subunits can still translocate DNA, suggesting that the subunits are not strictly coordinated^29^. If the motor subunits are not coordinated, slipping may occur when one subunit that is gripping the DNA releases its grip before another subunit forms a grip. Our finding that multiple ATP-bound subunits exert significant friction that minimizes slipping explains how the motor can prevent a significant length of DNA from exiting the capsid.

Our data also show that the packaging machine has a special mechanism that prevents the whole DNA molecule from exiting the capsid even if motor slipping occurs. It is quite striking that the clamped DNA is re-packaged into the head and such exiting/re-packaging events can be repeated multiple times. That this clamp is engaged regardless of the nucleotide state of motor subunits suggests that it may be mediated by another component of the packaging machine besides the motor protein. One possibility is that it is mediated by the portal protein (gp20), which forms the channel through which the DNA is translocated into the capsid. Indeed, this is plausible since it has been shown in both phage T4 and phage phi29 that the last-packaged section of the viral genome is tightly bound by the “tunnel loops” that are projected into the portal channel. Deletion of these loops causes release of some of the DNA when the motor undocks from the portal before the neck and tail of the virus are assembled^44,45^. Another possibility is that the first-packaged segment could be anchored inside the capsid. Consistent with this idea, fluorescence spectroscopy studies found evidence that both ends of the DNA are localized near the portion of the portal that extends into the capsid^46^. Further investigation of the mechanism would benefit from structural analysis, such as by imaging the complexes with cryo-electron microscopy.

We propose that the end clamp mechanism serves three important functions that are likely to be biologically relevant. First, at any stage of packaging, it would help prevent complete exit of the DNA if the ATP concentration transiently decreased, or for any other reason the motor slips. Second, during the early stages of packaging when only a small amount of DNA is inside, it would prevent exit of the DNA due to small slips that have been observed to occur even with saturating [ATP]^16^. Third, it would make initiation of packaging more efficient by making the process essentially irreversible. This would mitigate delays that would otherwise occur if the end of the DNA had to diffuse back into the motor channel. Minimizing the time that the DNA spends outside the capsid is advantageous because the DNA ends are highly susceptible to nucleases (e.g., RecBCD nuclease) present in the infected cell^47^.

## Methods

### Purification of phage capsids

The emptied phage capsids were purified from phage T4 *10am13am* mutant infected *E. coli* cells according to established methods^48^. Briefly, the infected cells were lysed with the Pi-Mg buffer (26 mM Na_2_HPO_4_, 68 mM NaCl, 22 mM KH_2_PO_4_, and 1 mM MgSO4, pH 7.5) containing 10 µg/ml DNAse I and a few drops of chloroform. The lysate was centrifuged at 4300×*g* for 10 min and the supernatant was centrifuged at 34,500×*g* for 45 min. The pellet containing the head particles was resuspended in 50 mM Tris-HCl and 5 mM MgCl_2_, pH 7.5 and subjected to low-speed and high-speed centrifugations as above. The head pellet was then resuspended in the same buffer and purified by CsCl density gradient centrifugation. The turbid band that contains empty heads was extracted from the side of the tube with a syringe and dialyzed overnight against 10 mM Tris-HCl, 50 mM NaCl, and 5 mM MgCl_2_, pH 7.5. The heads were then purified by ion-exchange chromatography using a DEAE-Sepharose column. The peak heads-containing fractions eluted by a 50–400 mM NaCl gradient were pooled and concentrated by an Amicon Ultra centrifugal filter (Millipore) and stored at −80 °C.

### Purification of gp17

The DNA packaging protein gp17 was purified according to established methods^49,50^. *E. coli* cells expressing N-terminally His-tagged recombinant gp17 were harvested by centrifugation at 3000×*g* for 10 min and the pellets were resuspended in 50 mM Tris-HCl buffer, pH 8.0, containing 5 mM imidazole. The cells were lysed by French Press and centrifuged at 28,000×*g* for 30 min. The supernatant was loaded onto a His-Trap column (GE Healthcare) pre-equilibrated with the same buffer. The His-tagged gp17 was eluted with a 5–250 mM imidazole gradient and the peak gp17-containing fractions were pooled and concentrated using an Amicon Ultra centrifugal filter (Millipore). The concentrated protein was purified by size exclusion column chromatography using a HiLoad 16/60 Superdex 200 prep-grade column (GE Healthcare) equilibrated with 50 mM Tris-HCl buffer, 5 mM MgCl_2_, and 100 mM NaCl, pH 8.0. The peak fractions were pooled and concentrated by Amicon Ultra centrifugal filter (Millipore) and stored at −80 °C.

### DNA substrate

The 10,051 bp biotin-labeled dsDNA was generated by PCR^39^ using the TaKaRa LA Taq kit (Takara Bio, Inc). The substrate DNA was lambda phage DNA (New England Biolabs) and the primers were as follows. “Lambda10kbForward”: (biotin TEG)-5′-CTGATGAGTTCGTGTCCGTACAACTGGCGTAATC and “Lambda10kbReverse”: 5′-ATACGCTGTATTCAGCAACACCGTCAGGAACACG (IDT DNA, Inc.).

### Single molecule assay

The optical tweezers instrument was calibrated by using DNA molecules as metrology standards^34,35^ and packaging measurements were conducted using established protocols^38,52^. In brief, T4 head-motor complexes were prepared by mixing 1.5 × 10^¬9^ heads with 75 picomoles of gp17 in a solution containing 50 mM Tris-HCl pH 7.5, 5 mM MgCl_2_, 80 mM NaCl, 1.5 mM γ-S-ATP, and 555 ng of 120 bp “initiating” DNA (which stabilizes the complexes) in a 13.5 μl reaction volume. This solution was incubated at room temperature for 45 min. One μl of anti-T4 antibody coated 2.2 μm diameter protein-G microspheres (Spherotech) was mixed with the T4 complexes and incubated at room temperature for another 30 min. One μl of rabbit antisera, containing polyclonal rabbit antibodies against the bacteriophage T4 capsid, was used per 5 μl of microspheres (5% w/v). The biotinylated DNA was attached to 2.2 μm diameter streptavidin coated microspheres (Spherotech). Measurements were carried out in solution containing 50 mM Tris-HCl pH 7.5, 5 mM MgCl_2_, 80 mM NaCl, and 0.05 g L^−1^ BSA. To initiate packaging, 1 mM ATP was added to the reaction mixture.

### Reporting Summary

Further information on experimental design is available in the Nature Research Reporting Summary linked to this article.

## Supplementary information

Supplementary Information

Reporting Summary

## Data Availability

The data that support the findings of this study are available from the corresponding authors upon reasonable request.
