# Supplementary material for: Nucleotide-dependent DNA gripping and an end-clamp mechanism regulate the bacteriophage T4 viral packaging motor
Source: Nat Commun. 2018 Dec 21;9:5434. doi: 10.1038/s41467-018-07834-2 (PMC6303390; doi:10.1038/s41467-018-07834-2)
Supplement: Supplementary file 1 — Supplementary Information [file 41467_2018_7834_MOESM1_ESM.pdf]

Supplementary Information

Nucleotide-dependent DNA gripping and an end-clamp mechanism regulate the bacteriophage T4 viral packaging motor

Ordyan et al.

Supplementary Note 1

Our model (Fig. 4) proposes that the DNA is gripped by a subunit which has nucleotide bound and slips when the nucleotide dissociates (or is hydrolyzed and the products dissociate). A standard type of model [1] assumes that such events occur with a constant probability per unit time at average rate k.

To investigate whether this model is consistent with the data we analyzed the durations of individual gripping events, which are predicted to follow an exponential distribution with mean duration $1/k$ [1]. Gripping events were identified using statistical criteria as follows. Velocities v in a 0.5 s sliding time window, slid in 0.01 s steps, were calculated by linear regression of the DNA length vs. time data and sections where $v<2\sigma_{\mathrm{control}}$ were scored. As explained in the text, $\sigma_{control}$ is the standard deviation in v measured in control experiments with a fixed length DNA tether and characterizes the effect of Brownian and instrumental noise on the measurement. For the 0.5 s time window $\sigma_{\mathrm{control}}$=20 bp s^{-1}. Shorter time windows were not used because $\sigma_{\mathrm{control}}$ increases rapidly with decreasing window size. Overlapping sections of data satisfying $v<2\sigma_{\mathrm{control}}$ were then merged to identify gripping events. A minor correction was also made since the velocity criterion alone allows small sections of slipping before and after each gripping event to be counted as part of the gripping event. To correct this, we calculated the mean length $\langle L\rangle$ and standard deviation σ_{L} during the middle half of each gripping event. Then, lengths smaller than $\langle L\rangle-2\sigma_{\mathrm{L}}$ on the left edge of the event and greater than $\langle L\rangle+2\sigma_{\mathrm{L}}$ on the right edge of the event were rejected as statistical outliers.

In this manner, individual gripping events were identified and a histogram of their durations for the 0.5 mM ADP condition was determined (Supplementary Fig. 1). Consistent with the model, this histogram is reasonably well fit by an exponential distribution, $\tau_1=C_1*exp(-k_1t)$, as shown in the figure, which yields k_1=3.5 s^{-1}.

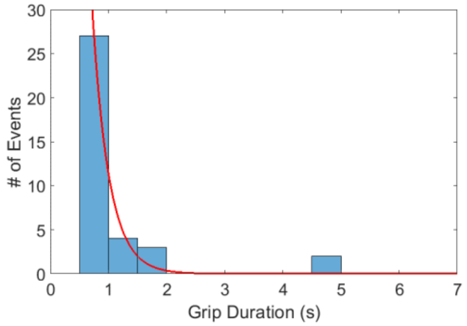

Supplementary Fig. 1. Histogram of the durations of gripping events measured with 0.5 mM ADP. Red line shows the fit to an exponential distribution.

Slipping events were identified as sections of data between identified gripping events and a histogram of the durations of the slipping events was also determined (Supplementary Fig. 2). These data were also reasonably well fit by an exponential distribution, $\tau_2=C_2*exp(-k_2t)$, as shown in the figure, which yields k_2=0.61 s^{-1}. Thus, transitions between slipping and gripping states can be characterized by the rates k_1 and k_2. Simulations of the model using these rates are presented in Supplementary Note 2.

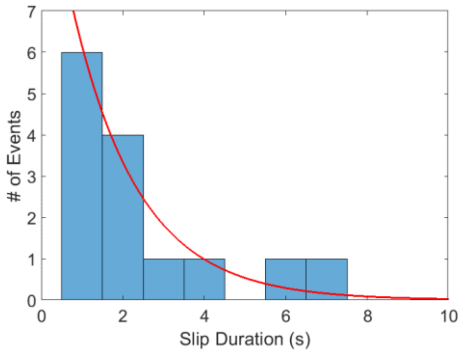

Supplementary Fig. 2. Histogram of the durations of slipping events measured with 0.5 mM ADP. Red line shows the fit to an exponential distribution.

We also analyzed the 0.17 mM ADP data. However, there is much less gripping in this condition. Only 6 gripping events were identified within the 27 datasets and only one dataset contained two gripping events (as needed to determine durations of slipping events). Thus, it was not feasible to determine distributions of durations of slipping and gripping events for this condition. However, the mean duration for the 6 gripping events measured with 0.17 mM ADP was 1.3 s (standard deviation=0.7) which is consistent with the mean duration of 1.1 s. (standard deviation=1) measured with 0.5 mM ADP. This finding is consistent with our model since the time for an ADP molecule to dissociate is not expected to depend on the concentration of ADP in the surrounding solution.

We also analyzed the γ-S-ATP data, but found that individual gripping events could not be reliably identified due to the Brownian and instrumental noise. Unlike with ADP, average slipping velocities with γ-S-ATP are very close to the velocity threshold used to discriminate gripping events from slipping events. With 0.5 mM γ-S-ATP the mean slipping velocity determined by the simple 1 s sliding window analysis is 34 bp s^{-1} (standard deviation=19 bp s^{-1}), which is only barely above the $2\sigma_{control}$=17 bp s^{-1} noise threshold for a 1 s sliding window and is below the $2\sigma_{control}$=40 bp s^{-1} noise threshold for the 0.5 s window used to detect gripping events in the ADP data analysis. Although individual gripping events could not be analyzed we emphasize that the conclusions made in the text regarding the behavior with γ-S-ATP remain well supported by the statistical analyses of the velocity distributions.

Supplementary Note 2

We present stochastic simulations of the model for the 0.5 mM ADP condition using the rate constants k_1 and k_2 determined above and calculation methods described in Ref. [1]. Durations of individual simulated gripping and slipping events were calculated by drawing values randomly from exponential distributions having mean values $\tau_i = 1/k_i$, using the "exprnd" function in Matlab (Version 2017b, Mathworks, Inc.).

The model in Fig. 4 also proposes that with ADP bound there are "intermediate" states in which the motor exerts varying levels of friction on the slipping DNA. These were proposed to explain the slipping velocities ranging from 34 bp s^{-1} (mean rate with γ-S-ATP) to ~2000 bp s^{-1} (rate observed with no nucleotides). In the simulations we assumed n=10 states (i=1, 2, 3, …10) having slipping velocities evenly spanning the range from 34 to 2000 bp s^{-1}. The v=2000 bp s^{-1} state corresponds to the "minimum friction" state in Fig. 4. The choice of n=10 was arbitrary, but

intended to account for the observation that slipping velocities span a wide range. Upon entering a slip we assumed that one of the states is entered at random. Transitions from state *i* to state *i+1* or *i-1* were assumed to occur with constant probability per unit time at an average rate of k_3=3 s^{-1}. A single transition rate was assumed for simplicity to minimize the number of adjustable model parameters. The value k_3=3 s^{-1} was chosen with the rationale it should be modestly higher than the value k_2=0.61 s^{-1}, so that the velocity would change several times during slips.

To account for the effect that Brownian motion and instrument noise has on the experimentally measured signal, we added a noise term to the simulated data that consisted of randomly chosen sections of noise fluctuations (instantaneous length minus mean length) recorded in the control measurements with fixed-length DNA molecules (see text).

Plots of the simulation results are shown in Supplementary Fig. 3. Comparison of these plots with the experimental results (Fig. 2 in the main text) shows that the predictions of the model are consistent with the experimentally observed behavior.

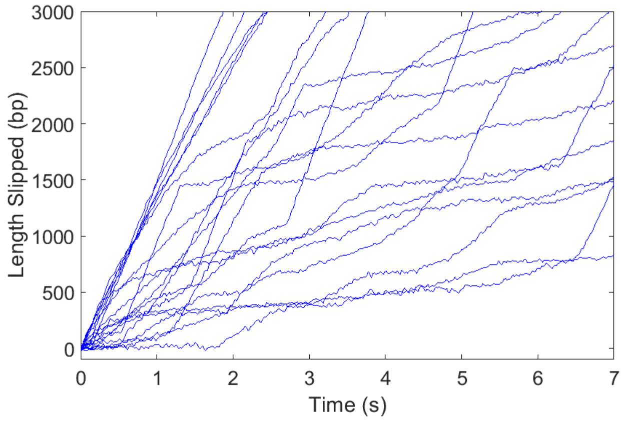

Supplementary Fig. 3. Plots of length of DNA slipped vs. time from stochastic simulations repeated 20 times.

Supplementary References

[1] R Erban, J Chapman, P Maini, "A practical guide to stochastic simulations of reaction-diffusion processes", arXiv preprint: https://arxiv.org/abs/0704.1908
